# In Vitro Antimicrobial Potential of Medicinal Plant Extracts and Their Combinations Against Mastitis-Causing Bacteria in Dairy Cows

**DOI:** 10.3390/molecules31071089

**Published:** 2026-03-26

**Authors:** Yuriy Balji, Leila Sultanayeva, Raikhan Mustafina, Meruyert Alyonova, Aleko Kalandia, Indira Djafaridze, Galia Zamaratskaia

**Affiliations:** 1 Institute of Animal Science and Veterinary Medicine, S.Seifullin Kazakh Agro Technical Research University, Astana 010000, Kazakhstan; yu.balji@kazatu.edu.kz (Y.B.); sultanaevaleila97@gmail.com (L.S.); r.mustafina@kazatu.edu.kz (R.M.); meruertalenova64@gmail.com (M.A.); 2 LLP “NFT-KATU”, Astana 010000, Kazakhstan; 3 Department of Chemistry, Faculty of Natural Sciences and Health Care, Batumi Shota Rustaveli State University (BSU), Batumi 6010, Georgia; aleko.kalandia@bsu.edu.ge (A.K.); indira.jafaridze@bsu.edu.ge (I.D.); 4 Department of Molecular Sciences, Swedish University of Agricultural Sciences, 750 07 Uppsala, Sweden

**Keywords:** bovine mastitis, phytobiotics, antimicrobial activity, phenolic compounds, flavonoids, *Staphylococcus aureus*, *Escherichia coli*, *Candida albicans*

## Abstract

Mastitis in dairy cows, caused by pathogens such as *Staphylococcus aureus*, *Escherichia coli*, and *Candida albicans*, leads to substantial economic losses and contributes to antimicrobial resistance, emphasizing the need for natural alternatives. This study assessed the phytochemical composition, antioxidant activity, and in vitro antimicrobial effects of ethanol extracts from ten medicinal plants, including *Populus balsamifera* buds, *Syzygium aromaticum*, and *Humulus lupulus*, as well as two multi-component plant mixtures and commercial products against reference strains and field isolates of mastitis pathogens. Extracts exhibited total phenolic contents ranging from 2.5 to 43.0 mg GE/g, with *Populus balsamifera* and *Syzygium aromaticum* demonstrating the strongest DPPH radical scavenging activity (IC_50_ 1.89–2.9 mg/g extract). Disc diffusion assays demonstrated broad-spectrum inhibition, particularly for *Populus balsamifera* (15.1–19.1 mm inhibition zones) and *Humulus lupulus* (9.0–18.4 mm) against key pathogens; phenolic and flavonoid contents positively correlated with antimicrobial activity (r = 0.63–0.99, *p* < 0.001). Multi-component mixtures provided consistent broad-spectrum effects (12.6–17.2 mm). These phenolic-rich plant extracts represent promising alternatives to reduce antibiotic use in dairy mastitis management.

## 1. Introduction

Mastitis in dairy cows remains one of the most common and economically significant mammary gland infections worldwide. It leads to reduced milk yield, deterioration of milk quality, increased culling rates, and high treatment costs [1]. Clinical and subclinical forms are associated with elevated somatic cell counts and microbial contamination of milk, which directly impact processing and food safety [2]. The etiologic spectrum of mastitis in dairy cows includes *Staphylococcus aureus*, *Escherichia coli*, *Streptococcus agalactiae*, and other streptococci and coagulase-negative staphylococci (CNS) [3]. Growing evidence suggests that *Staphylococcus aureus* can persist intracellularly, promoting chronic and recurrent mastitis [4]. Moreover, these pathogens often develop resistance to multiple classes of antimicrobial drugs, complicating therapy and increasing the risk of treatment failure [5]. Inappropriate use of antibiotics in cattle production increases antimicrobial resistance (AMR) and leads to the presence of drug residues in raw milk and dairy products [6]. These residues have been associated with alterations in gut microbiota composition, the selection of antibiotic-resistant bacteria, and hypersensitivity reactions [7,8,9]. In some cases, these residues exceed maximum residue limit (MRL). For example, a recent review of raw cow’s milk reported that β lactams and tetracyclines were among the most frequently detected antibiotic residues, and in several studies, concentrations were above legal MRLs [10]. In a field study from Algeria, over half (55.3%) of tested raw milk samples exceeded the MRL for β lactams, with very high levels of cloxacillin and penicillin G [11]. Poor adherence to withdrawal periods, the time required between treatment and milk collection, is a likely contributing factor and has been documented in livestock systems [12]. Thus, there is a growing interest in safer, natural alternatives for preventing and treating mastitis in dairy cows. Among these, plant-derived extracts and phytobiotics are promising alternatives due to their antimicrobial and anti-inflammatory properties [13,14]. For example, thyme essential oil demonstrated strong bactericidal activity against major mastitis pathogens in milk [15]. Another promising approach is the co-use of essential oils with medium-chain fatty acids. In vitro, carvacrol in combination with octanoic acid achieved over 6-log reduction in typical mastitis pathogens and disrupted their cell membranes [16]. There is also in vivo evidence. An essential-oil-based formulation (Phyto-Bomat) has been tested in dairy cows with mastitis, where it showed antifungal activity against yeast in milk [17]. Also, a natural phytotherapeutic mix containing propolis, hop extracts, lemon balm, and other plant components reduced bacterial counts in mastitic cows in a small trial [18]. A plant-extract-based teat dip (papain, aloe, and other botanicals) significantly lowered microbial load on teats under commercial farm conditions [19].

This study aimed to identify promising plant-based candidates for alternative antimicrobial strategies against key mastitis pathogens in dairy cattle. For this purpose, we evaluated the phytochemical composition and in vitro antimicrobial activity of ethanol extracts from 10 selected medicinal plant species (*Populus balsamifera*, *Syzygium aromaticum*, *Artemisia absinthium*, *Plantago major*, *Achillea millefolium*, *Humulus lupulus*, *Fomes fomentarius*, *Coriandrum sativum*, *Silybum marianum*, and *Matricaria chamomilla*), as well as selected combinations of these extracts. The plant species included in this study were selected based on several criteria. First, these plants have traditionally been used in Kazakh folk veterinary medicine for the treatment of mastitis and wound healing. Second, the selected species are widely distributed and readily available in the regions where the plant materials were collected. In addition, the selection was supported by scientific reports indicating that these plants contain bioactive compounds, such as phenolics and essential oils, which have been shown to exhibit antimicrobial activity against mastitis-associated pathogens [13,14,15,16,19].

## 2. Results

### 2.1. Phytochemical Composition and Antioxidant Activity of Plant Extracts

Significant differences in phytochemical profiles and antioxidant capacity were observed among the ethanol extracts of the investigated medicinal plant species (Table 1). Extracts from balsam poplar buds and clove were characterized by higher concentrations of phenolic constituents compared with the other samples, and this was reflected in their pronounced radical scavenging activity. In contrast, extracts such as coriander, hops, and polypore exhibited substantially lower levels of phenolic compounds and correspondingly weaker antioxidant effects. Phenolic acids and flavonoids constituted the dominant phenolic fractions across most extracts, whereas catechins were present at very low levels or below the detection limit in the majority of samples. Measurable catechin content was detected only in a limited number of extracts and at comparatively low concentrations.

UPLC–PDA–MS analysis of *Artemisia absinthium* extract identified thirteen phenolic compounds, dominated by hydroxycinnamic acid derivatives. Two caffeoylquinic acid isomers (*m*/*z* ≈ 353) and two dicaffeoylquinic acid isomers (*m*/*z* ≈ 515) were detected as the major constituents, alongside feruloylquinic acid derivatives (*m*/*z* ≈ 363) and a flavone glycoside tentatively assigned to a luteolin-type structure (*m*/*z* ≈ 563). Minor compounds included a methoxycinnamic acid derivative (*m*/*z* ≈ 293) and several unidentified phenolic conjugates.

Analysis of *Populus balsamifera* bud extract revealed a complex phenolic profile comprising hydroxycinnamic acids, flavanones, flavones, flavonols, and salicylate-type phenolic glycosides. The dominant compounds included pinobanksin-type flavanones (*m*/*z* ≈ 271), chrysin (*m*/*z* ≈ 263), a galangin-type flavonol (*m*/*z* ≈ 270), acacetin (*m*/*z* ≈ 297), and pinocembrin-like flavanones (*m*/*z* ≈ 267). A peak at *m*/*z* 537 was tentatively identified as tremulacin, a salicylate glycoside characteristic of the genus Populus. This combination of compounds constitutes a characteristic Populus-type flavonoid fingerprint.

In the *Humulus lupulus*, the chromatographic profile was dominated by caffeoylquinic acid derivatives (*m*/*z* ≈ 353), including quinic acid (*m*/*z* 191.02) as a structural component. Two peaks at *m*/*z* ≈ 579 were attributed to procyanidin dimers, while additional flavonoid-related compounds were detected, including a luteolin-type flavone glycoside (*m*/*z* ≈ 563), a kaempferol derivative (*m*/*z* ≈ 285), and a coumaroylquinic acid derivative (*m*/*z* ≈ 329).

**Figure 1:**
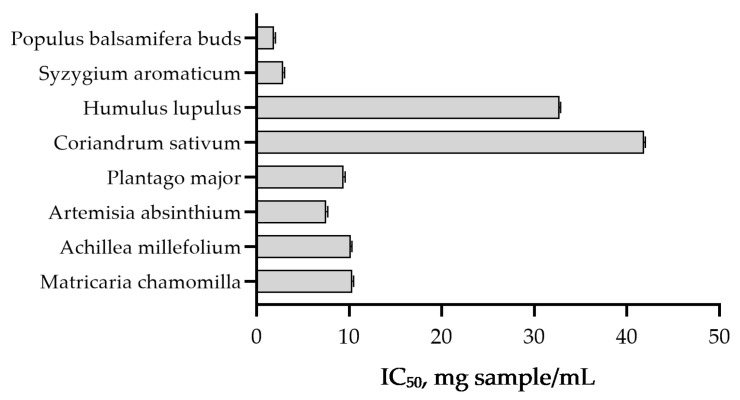
DPPH radical scavenging activity of tested samples. Antioxidant activity is expressed as IC_50_ (mg of sample required for 50% DPPH radical inhibition). Data are presented as LSMeans ± SE of three replicates. Lower IC_50_ values indicate stronger antioxidant capacity. *Silybum marianum* and *Fomes fomentarius* did not reach 50% inhibition within the tested concentration and are not presented in the figure.

Antioxidant activity, expressed as IC_50_ of DPPH radical scavenging, differed significantly among all plant extracts (*p* < 0.001). *Populus balsamifera* buds exhibited the strongest antioxidant activity, with the lowest IC_50_ value (1.89 mg/g extract), followed by *Syzygium aromaticum* (2.9 mg/g extract) (Figure 1). Moderate radical scavenging capacity was observed for *Artemisia absinthium* (7.5 mg/g extract), *Plantago major* (9.4 mg g^−1^ extract), and *Achillea millefolium* (10.2 mg/g extract). Higher IC_50_ values, indicating lower antioxidant activity, were recorded for *Humulus lupulus* (32.7 mg/g extract) and *Coriandrum sativum* (41.8 mg/g extract). Extracts of *Silybum marianum* and *Fomes fomentarius* did not reach 50% inhibition and were therefore considered inactive under the applied experimental conditions.

Pearson correlation coefficients (r) between phytochemical contents and antimicrobial inhibition zones are presented in Table 2. Positive correlations (r > 0) indicate that higher concentrations of certain phytochemical groups were associated with larger inhibition zones against specific pathogens. For instance, total phenolics and flavonoids showed significant positive correlations with inhibition of *Candida* field isolates (r = 0.848 and r = 0.731, respectively; *p* < 0.05), suggesting a potential association between these compounds and antifungal activity.

Additional correlations included positive relationships between total phenolics, flavonoids, and catechins with inhibition of total field isolates, as well as between catechin content and inhibition of *S. aureus* ATCC. In contrast, a negative correlation was observed between phenolic acids and inhibition of *E. coli* field isolates. No statistically significant correlations were observed between IC_50_ values and total phenolics, phenolic acids, flavonoids, or catechins (*p* > 0.05) (Table 2).

### 2.2. Antimicrobial Data

Plant extracts demonstrated moderate-to-strong antimicrobial activity, with *Populus balsamifera* (15.1 mm *Candida* field, 19.1 mm *S. aureus*) and *Humulus lupulus* (18.2 mm *Candida* field, 18.4 mm *S. aureus*) achieving the largest zones against key mastitis pathogens (Table 3). *Syzygium aromaticum* excelled against *Candida* ATCC (14.5 mm) and *E. coli* ATCC (17.5 mm). Plantago major showed the strongest activity against field *Candida* (8.1 mm) but had inconsistent performance across strains. *Humulus lupulus* provided broad-spectrum inhibition across most targets (9.0–18.4 mm). Formulations and commercial products varied: CP2 peaked against *Candida* ATCC (28.8 mm), F2 showed broad activity (12.6–17.2 mm), while F1 had limited effects against field isolates (3.2–7.6 mm). Reference strains were generally more susceptible than field isolates, reflecting greater resistance in clinical mastitis pathogens. Two-way ANOVA confirmed significant effects of extract (*p* < 0.001), microbe (*p* = 0.001), and the interaction (*p* < 0.001). All extracts and formulations exceeded ethanol control (Dunnett *p* < 0.001).

**Figure 2:**
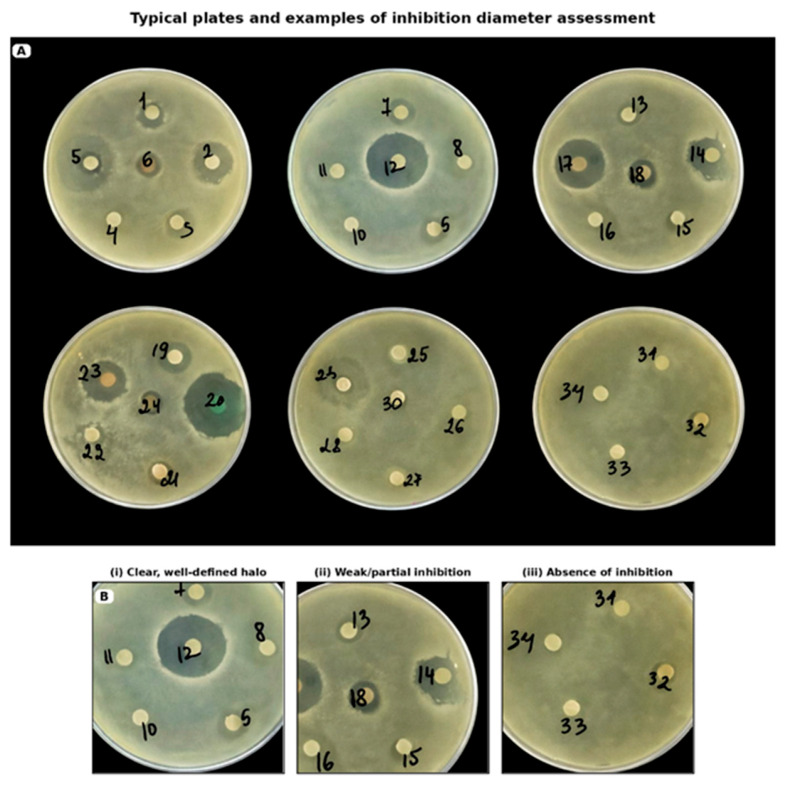
Disc diffusion assay showing the antifungal activity of selected samples against a production strain of *Candida albicans*. (**A**) Representative Petri dishes used for the assessment of inhibition zones for the tested samples. The numbered discs correspond to the following samples: 1—*Fomes fomentarius*; 2—*Coriandrum sativum*; 5—*Matricaria chamomilla*; 7—*Plantago major*; 11—*Achillea millefolium*; 12—*Populus balsamifera* buds; 14—*Artemisia absinthium*; 17—*Syzygium aromaticum*; 19—F1; 20—F2; 23—*Humulus lupulus*. (**B**) Representative examples of inhibition zone assessment: (i) clear, well-defined halo; (ii) weak/partial inhibition; (iii) absence of inhibition.

**Figure 3:**
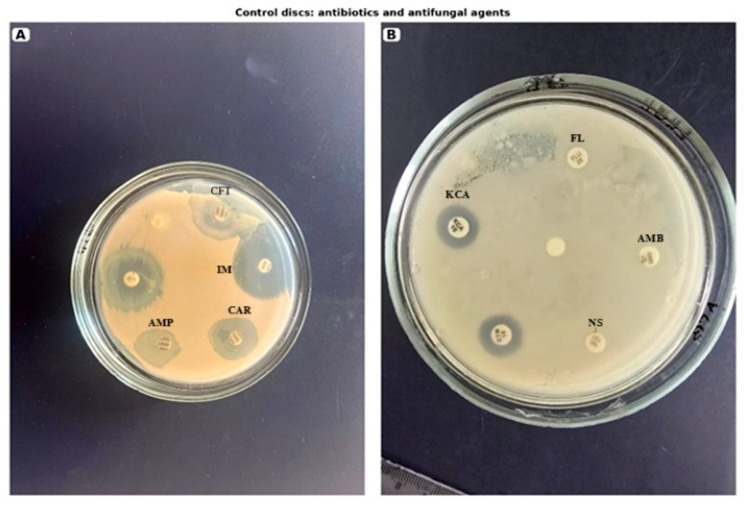
Representative images of disc diffusion assay plates with control antibacterial and antifungal discs used against the tested microorganism. (**A**) Antibacterial control discs: CFT, cefalotin; AMP, ampicillinum; CAR, carbenicillin, IM, imipenem. (**B**) Antifungal control discs: FL, fluconazole; AMB, amphotericin B; NS, nystatin; KCA, ketoconazole.

Representative disc diffusion plates are presented in Figure 2 and Figure 3.

The control antimicrobial drugs exhibited selective activity against the tested microorganisms. Among the antifungal agents, ketoconazole inhibited the production strain of Candida (8.9 ± 2.9 mm), whereas fluconazole and nystatin showed no inhibitory effect against either Candida strain under the tested conditions (0.0 ± 2.9 mm). Among the antibacterial agents, imipenem and carbenicillin inhibited the production strain of *Escherichia coli*, with inhibition zones of 11.6 ± 2.9 mm and 10.5 ± 2.9 mm, respectively. In addition, ampicillinum showed activity against the “Total field” production strain (14.5 ± 2.9 mm), while cefalotin showed no detectable effect (0.0 ± 2.9 mm).

## 3. Discussion

The complete elimination of antibiotics in livestock is currently unfeasible, particularly for acute infections that require rapid control of pathogens. However, integrating phytobiotics and phytogenics into treatment protocols can enhance the efficacy of conventional drugs, reducing both dosage and duration, and is particularly valuable for chronic infections such as bovine mastitis, where prolonged antibiotic use may drive resistance. Phytobiotics also offer prophylactic benefits, strengthening immunity, improving gut microbiota, and lowering infection risk. For example, ethanolic pomegranate (*Punica granatum* L.) peel extracts (25–50 mg/g extract) exhibit rapid bactericidal activity against *E. coli* and *S. aureus*, comparable to commercial disinfectants, via membrane disruption [20]. Similarly, several plant extracts used against bovine mastitis pathogens (e.g., *Plectranthus ornatus*, *Salvia officinalis*, *Senna macranthera*) displayed clear synergism with ampicillin, gentamicin, and other mastitis antibiotics, allowing up to 8-fold reductions in the minimum inhibitory concentrations [21,22].

The pronounced differences in radical scavenging capacity observed across the investigated extracts can be interpreted in light of their phytochemical profiles, since phenolic compounds, particularly flavonoids and phenolic acids, are widely recognized as the primary mediators of antioxidant activity in plant-derived materials [23]. This relationship has been consistently demonstrated across diverse plant species. Strong positive correlations between total phenolic content, total flavonoid content, and DPPH radical scavenging activity have been documented in numerous studies [24,25,26]. In the present study, individual phenolic constituents were tentatively identified by UPLC–PDA–MS based on mass spectral and UV characteristics; however, absolute quantification was not performed because authentic reference standards were not available for all detected compounds. Therefore, the chromatographic analysis was used primarily to characterize the qualitative phenolic profiles of the investigated extracts. Flavonoids such as quercetin, apigenin, luteolin and epigallocatechin derivatives have been shown to interact with antibiotics to lower minimum inhibitory concentrations and improve bacterial inhibition against a range of Gram-positive and Gram-negative pathogens, including resistant strains, by mechanisms such as increased membrane permeability and efflux pump modulation [27,28]. Moreover, plant extracts rich in phenolics have restored antibiotic susceptibility in multidrug-resistant *Staphylococcus aureus* and *Acinetobacter baumannii* when combined with ampicillin and ciprofloxacin, highlighting the adjuvant potential of plant bioactives in antimicrobial therapy [27]. These findings support our observations of broad-spectrum inhibition and underscore the potential of flavonoid-rich extracts to both complement antibiotic action and contribute to multifaceted antimicrobial strategies.

The antimicrobial performance observed in the present study can be partly interpreted in light of the quantitative differences in phenolic composition among the investigated extracts. *Populus balsamifera* buds and *Syzygium aromaticum*, which exhibited the highest total phenolic and flavonoid concentrations, also demonstrated strong antioxidant activity and broad antimicrobial effects, including pronounced inhibition of *Candida* spp. and *S. aureus*. Correlation analysis further supported this relationship, revealing positive associations between total phenolics, flavonoids, and inhibition of selected microbial targets. These findings are consistent with previous reports indicating that phenolic-rich extracts exert antibacterial effects through membrane disruption, enzyme inhibition, metal chelation, and interference with virulence factors such as biofilm formation and quorum sensing [21]. The comparatively weaker activity of extracts with lower phenolic content, such as *Coriandrum sativum* and *Fomes fomentarius*, further supports the role of phenolic constituents as major contributors to the observed antimicrobial efficacy.

The present study demonstrated that antimicrobial activity was significantly associated with some specific phenolic subclasses, particularly flavonoids and catechins, whereas antioxidant capacity (IC_50_) showed no direct relationship with microbial inhibition. These findings are consistent with previous reports indicating that the relationship between antioxidant activity and antimicrobial potency is variable and may be weak or absent depending on the tested microorganism [29]. This dissociation is expected, as flavonoids exert membrane-disrupting effects through mechanisms independent of their radical scavenging capacity [30].

The antimicrobial activity observed across the plant extracts and formulations in this study reflects diverse phytochemical mechanisms that have been documented in previous research. Extracts from *Populus balsamifera* buds exhibited large inhibition zones against both Gram-positive (*S. aureus*) and Gram-negative (*E. coli*) mastitis pathogens [31,32]. *Syzygium aromaticum* essential oils inhibited *S. aureus* mastitis strains and showed synergy with antibiotics against Gram-negative bacteria [33]. These species have been previously characterized for high levels of phenolic acids and flavonoids, which correlate with antimicrobial effects against mastitis isolates. In particular, *Syzygium aromaticum* exhibited a wide range of biological activities due to its chemical constituents, including total phenolic compounds and flavonoids. Its essential oil, with eugenol as the major component (53%), has demonstrated antimicrobial, antioxidant, and antitrypanosomal effects, and showed higher activity against *Staphylococcus aureus* than eugenol alone and low cytotoxicity [34]. *Humulus lupulus* also demonstrated true broad-spectrum inhibition across all tested pathogens in the present study. This broad-spectrum profile is particularly valuable from a practical standpoint, since mastitis in dairy cows often involves mixed pathogen populations rather than a single organism. The antimicrobial activity of *Humulus lupulus* is primarily attributed to its hop bitter acids, alpha-acids (humulones) and beta-acids (lupulones), as well as the prenylated flavonoid xanthohumol, which has been shown to disrupt bacterial cell membranes and interfere with proton transport [35,36]. Notably, hop-derived compounds have shown synergistic effects with conventional antibiotics against both Gram-positive and Gram-negative bacteria [37].

F2, which contained higher-activity individual extracts (*Syzygium aromaticum* and *Populus balsamifera* buds alongside *Coriandrum sativum*, *Fomes fomentarius*, and *Matricaria chamomilla*), achieved consistently broad-spectrum inhibition, which indicates that combining phenolic-rich extracts with complementary phytochemical profiles can yield reliable activity across multiple pathogen types. This is consistent with the previous results showing that multi-component plant-derived formulations can outperform individual extracts, likely due to synergistic interactions among bioactive compounds acting on multiple bacterial targets simultaneously [38]. In contrast, F1 showed limited inhibitory effects against field isolates, despite containing several individually active extracts including *Humulus lupulus*, *Populus balsamifera* buds, and *Plantago major*. This weaker performance may be explained by the higher water and glycerin content of F1 compared to F2, which would reduce the effective concentration of bioactive compounds delivered to the disc. It can also be explained by possible antagonistic interactions among the component mixture [39]. The broader antimicrobial activity observed for F2, compared with several individual extracts, may indicate additive or complementary interactions among its constituent plant extracts. Similar effects have been reported for multi-component phytopreparations, in which different classes of bioactive compounds, including phenolics and essential oils, may act on distinct microbial targets. Although our study was not specifically designed to quantify synergy, the performance of F2 supports further targeted evaluation of combinatory effects between its constituent plant extracts.

Regarding commercial preparations, CP2 showed the largest inhibition zone recorded in the entire study (28.8 mm against Candida ATCC), substantially exceeding all plant extracts tested. However, this activity appeared highly selective, as CP2 showed only moderate effects against other pathogens. CP1 demonstrated moderate activity against field isolates. Neither commercial preparation contained plant extracts, and their mechanisms of action differ fundamentally from those of phenolic-rich plant extracts. The strong antifungal activity of CP2 may reflect the known antifungal properties of citric acid and sorbitol-based formulations, which can disrupt fungal cell membrane integrity and reduce pH below the growth optimum of *Candida* spp. [40]. Importantly, the consistently strong and broad-spectrum activity of F2 across both reference strains and field isolates compares favorably with the commercial products.

Thus, our results strongly indicated the potential of selected phenolic-rich plant extracts as promising candidates for further development as complementary strategies in mastitis management, particularly in the context of reducing antibiotic reliance and mitigating antimicrobial resistance risks in dairy production systems.

A methodological limitation of this study is that herbarium voucher specimens were not formally deposited, although detailed taxonomic documentation (Table 4 and Table 5) and collection photographs (Supplementary Figures S1–S10) ensure material traceability.

## 4. Materials and Methods

### 4.1. Plant Materials

The characteristics of the plant and fungal materials used in the study are summarized in Table 4 and Table 5, respectively. Botanical identification was performed using the international database Plants of the World Online (Royal Botanic Gardens, Kew (Richmond, UK) [41], the State Pharmacopoeia of the Republic of Kazakhstan (SP RK) [42], and State Pharmacopoeia of the Russian Federation [43] (for *Humuli lupuli fructus*, *Syzygium aromaticum* (L.) Merr. & L.M.Perry and *Populus balsamifera* L.). Taxonomic verification of the plant material was carried out by specialists from the Institute of Agriculture and Forestry, Candidate of Biological Sciences Damilya Töremuratovna Konysbayeva and PhD Klara Serzhanovna Izbastina.

*Coriandrum sativum* (manufactured in the Russian Federation) and *Syzygium aromaticum* Merr. & L.M.Perry (manufactured in the UAE) were purchased from a retail chain in Astana, Kazakhstan. The raw materials purchased were in full conformity with TR CU 021/2011 “On Food Safety” [44] and TR CU 022/2011 “On the Labelling of Food Products” [45], as established by the Eurasian Economic Union.

Medicinal plant samples were collected monthly from three regions of Kazakhstan, Almaty region (Talgar district), Zhetysu region (Alakol district), and Akmola region, over the full vegetation cycle (June 2024–June 2025). Collections followed Good Agricultural and Collection Practices (GACP) as recommended by the World Health Organization and were limited to no more than 20% of the plant population. Species were selected based on two main criteria: documented ethnoveterinary use for udder health in Kazakh traditional veterinary practice and literature reports indicating the presence of bioactive compounds, such as phenolics and essential oils, with reported antimicrobial or anti-inflammatory activity relevant to mastitis-associated pathogens. Plant material was collected during the mass flowering phase (08:00–10:00 a.m.) and identified based on morphological characteristics according to Pavlov (1956–1966) [46]. Although comprehensive identification details are provided (Table 4 and Table 5, Supplementary Figure S1), formal herbarium deposition and voucher numbers were not completed during this study.

The samples were shade-dried at 20–35 °C with good ventilation for 48 h until completely air-dried and then ground with a hammer mill (LLP “Agrotechservis”, Kostanay, Kazakhstan) with a power of 0.75 kW prior to extraction. Ten plant species were used in this study. The following plant parts were collected and processed for extraction: buds of *Populus balsamifera*, flower buds of *Syzygium aromaticum*, aerial parts of *Artemisia absinthium*, leaves of *Plantago major*, flowering aerial parts of *Achillea millefolium*, strobiles (cones) of *Humulus lupulus*, basidiocarps of *Fomes fomentarius*, seeds of *Coriandrum sativum*, seeds of *Silybum marianum*, and flower heads, stems, and leaves of *Matricaria chamomilla*. The basidiocarps of the polypore fungus were collected from the trunks of *Populus balsamifera* trees in the forest zone of the Akmola region between March and October 2025. The species was identified as *Fomes fomentarius* based on morphological characteristics [47]. Basidiocarps were manually removed from the substrate using a knife. The collected basidiocarps were cleaned, and the hymenophore portions were separated, cut into small pieces, and air-dried in the shade for 48 h at room temperature. Subsequently, the samples were dried to constant weight in a drying oven (SNOL 24/200 LSP01, SNOL, Utena, Lithuania) at 40 °C for 72 h to prevent thermal degradation of biologically active compounds. The dried material was ground into a fine powder using a mill, sieved through a 0.5 mm mesh, and stored in airtight, light-protected containers at 4 °C until extraction.

### 4.2. Extraction

Air-dried plant material was ground to a fine powder using a hammer mill (LLP “Agrotechservis”, Kostanay, Kazakhstan; 0.75 kW) and subsequently passed through a 0.5 mm mesh sieve to ensure uniform particle size.

Hot extraction was performed with 70% aqueous ethanol (*v*/*v*) at a sample-to-solvent ratio of 1.5:10 (*w*/*v*). Briefly, 15 g of dried plant powder was placed into a cellulose extraction thimble and extracted with 100 mL of 70% ethanol in a Soxhlet apparatus. The extraction was carried out at 70 °C with continuous solvent circulation for 6 h (approximately 6–7 siphon cycles per hour), ensuring exhaustive extraction of bioactive compounds

After extraction, the solutions were divided into two portions: one for analysis of phenolic compounds, and the other concentrated using a rotary evaporator at 70 °C to one-third of the initial volume.

The dry residue mass of each extract was determined gravimetrically according to the European Pharmacopoeia (2022, § 2.8.16) [48]. Briefly, 1 mL of extract was placed in a pre-weighed aluminum dish and dried in an oven at 105 °C for 3 h until constant weight. The dry residue content was calculated as a percentage (%) and subsequently converted to mg of dry weight per mL of extract (mg DW/mL).

### 4.3. Formulations

Two plant extract formulations (F1 and F2) and two commercial preparations (CP1 and CP2), as well as an ethanol control, were also tested. F1 and F2 were prepared on a water–glycerin base containing standardized plant extracts and xanthan gum as a thickening agent (Table 6). F1 was prepared as a water–glycerin-based formulation containing several traditionally used medicinal plant extracts to provide a broad phytochemical composition suitable for routine teat application. F2 was formulated as a more concentrated preparation enriched in phenolic-rich extracts (e.g., *Populus balsamifera*, *Syzygium aromaticum*) and supplemented with flax oil and beeswax to improve delivery to the teat surface and enhance formulation stability. The formulation was designed to provide a higher concentration of phenolic-rich extracts while maintaining suitable physicochemical properties for topical application. CP1 was a commercially available preparation containing sodium laureth sulfate, glycerin, lactic acid, urea, anionic surfactants, eucalyptus fragrance, and demineralized water. CP2 was a commercial preparation composed of sodium citrate, citric acid, sorbitol, glycerin, xanthan gum, and demineralized water.

### 4.4. Spectrophotometric Quantification of Phenolic Compounds

Total phenolic content (TPC) was determined using the Folin–Ciocalteu colorimetric method [49], with minor modifications. Briefly, 1.0 mL of appropriately diluted extract was mixed with 1.0 mL of Folin–Ciocalteu reagent and incubated at room temperature for 3 min. Subsequently, 1.0 mL of 10% (*w*/*v*) sodium carbonate (Na_2_CO_3_) solution was added. The reaction mixture was diluted to a final volume of 10 mL with distilled water and thoroughly mixed. The samples were incubated in the dark at room temperature for 90 min to allow full color development. Absorbance was measured at 750 nm using a UV-Vis spectrophotometer (Mettler-Toledo International Inc., Columbus, OH, USA). against a reagent blank. Quantification of total phenolic content was performed using gallic acid as the reference standard. A calibration curve was prepared using gallic acid solutions at different concentrations, and the absorbance values were measured under identical conditions to those of the samples. The calibration curve showed excellent linearity and was described by the regression equation y = 0.9524x − 0.0084 (R^2^ = 0.9996), where *y* represents the absorbance and *x* represents the concentration of gallic acid. Total phenolic content was calculated using the calibration curve and expressed as milligrams of gallic acid equivalents per gram of extract (mg GAE g^−1^ extract)**.**

Total phenolic acid content was determined spectrophotometrically at 320 nm using a spectrophotometer (Mettler-Toledo International Inc., Columbus, OH, USA). Briefly, 250 µL of extract was mixed with 250 µL of 0.1% ethanolic HCl and 4.55 mL of 2% HCl (final volume 5.05 mL), vortexed, and incubated for 15 min at room temperature, and the absorbance was measured at 320 nm against a reagent blank. The concentration was calculated from the calibration curve (y = 4.1338x + 0.0393, R^2^ = 0.9986) and expressed as mg caffeic acid equivalents per mL of extract (mg GAE g^−1^ extract), taking into account the dilution factor [50].

Total flavonoid content (TFC) was determined by the AlCl_3_ method. Briefly, 1 mL of extract was mixed with 5 mL of distilled water and 0.3 mL of 5% NaNO_2_ and incubated for 5 min, followed by the addition of 0.3 mL of 10% AlCl_3_ (6 min incubation) and 2 mL of 1 N NaOH. Absorbance was measured at 510 nm, and results were calculated from a quercetin standard curve (y = 1.1543x − 0.0023, R^2^ = 0.9992) and expressed as mg quercetin equivalents per mL of extract (mg GAE g^−1^ extract) [50].

Total catechin content (TCC) was assessed using the vanillin assay. Briefly, 1 mL of extract was mixed with 3 mL of vanillin reagent, and the absorbance of the developed red color was measured at 500 nm after 3 min. A blank containing an extractant instead of a sample was included. Quantification was performed using a (+)-catechin standard curve (y = 33.615x − 0.0364, R^2^ = 0.9952), and results were expressed as mg catechin equivalents per mL of extract (mg GAE g^−1^ extract) [51].

### 4.5. UPLC–PDA–MS Profiling of Individual Phenolic Compounds

Individual phenolic compounds in the plant extracts were identified using Ultra Performance Liquid Chromatography coupled with photodiode array and mass spectrometry detection (UPLC–PDA–MS) on a Waters Acquity H-Class system equipped with a Quaternary Solvent Manager, Sample Manager–FTN, PDA detector, and QDa mass detector. Chromatographic separation was performed on a BEH C18 column (1.7 μm particle size) using a binary mobile phase consisting of 0.2% formic acid in water (solvent A) and acetonitrile (solvent B). Gradient elution was applied as follows: starting at 5% B, increased to 95% B over 10 min, held at 95% B until reaching 11 min, returned to 5% B at 12 min, followed by a 3 min equilibration. The flow rate was set at 0.3 mL min^−1^ and the column temperature was maintained at 40 °C. Mass spectrometric detection was carried out in negative electrospray ionization (ESI) mode with a scan range of 100–1200 Da. Phenolic compounds were identified based on retention time, UV–Vis spectral characteristics, and deprotonated molecular ions ([M − H]^−^), as well as comparison with previously published data. The same chromatographic conditions were applied for the analysis of all investigated plant extracts [52]. For detailed UPLC–PDA–MS profiling, we selected *Populus balsamifera*, *Artemisia absinthium*, and *Humulus lupulus* as a representative subset with contrasting phenolic levels and bioactivity.

### 4.6. Antioxidant Activity

Antioxidant activity was evaluated by the DPPH radical scavenging assay [53]. Briefly, 1.0 mL of extract at different concentrations was mixed with 3.0 mL of freshly prepared DPPH solution and incubated in the dark at room temperature for 15 min. The absorbance was then measured at 517 nm against an appropriate blank. A control sample containing the DPPH solution without extract was used for comparison.

The radical scavenging activity was calculated using the following equation:

Inhibition (%) = [(Acontrol − Asample)/Acontrol] × 100

where Acontrol is the absorbance of the control and Asample is the absorbance in the presence of the extract.

The IC_50_ value was calculated using the following equation:

IC_50_ = (m × V × F × 100)/In%50

where m is the mass of the tested sample (mg), V is the volume of the sample extract (mL), F is the dilution factor, and In%50 represents the percentage of DPPH inhibition within the 45–55% range used to interpolate the IC_50_ value. Results were expressed as IC50 (mg/g extract). Lower IC_50_ values indicate stronger radical scavenging activity. All analyses were performed in triplicate.

### 4.7. Microorganisms, Nutrient Media, and Cultivation Conditions

The study used deposited microbial strains, namely *Staphylococcus aureus* ATCC 6538 (RKM 0470), *Escherichia coli* ATCC 25922 (RKM 0447), and *Candida albicans* ATCC 885-653 (RKM 0475), obtained from the Republican Collection of Microorganisms LLP (Astana, Kazakhstan). Prior to testing, *S. aureus* and *E. coli* cultures were grown on Mueller–Hinton agar and Endo agar, respectively, at 37 °C for 24 h, and C. albicans was cultured on Sabouraud dextrose agar at 24 °C for 24 h. Culture purity was confirmed by microscopy (Gram staining) and by plating on Compact Dry selective/chromogenic media.

To obtain field isolates on a farm in the Akmola region (Kazakhstan), animals were divided into three groups based on comparative analysis. Group I included cows with subclinical mastitis, group II included cows with clinical mastitis, and Group III included healthy cows. Subclinical mastitis was detected using the California Mastitis Test (CMT). Teat skin swabs (lateral surface and sphincter area) were collected before and after milking using a Promedia ST-25 swab system (10 mL transport medium).

The microbiological bacterial load of milk was determined in the food safety laboratory of the Kazakh Agrotechnical Research University and at the Republican State Enterprise on the Right of Economic Management “Center for Sanitary and Epidemiological Expertise” of the Medical Centre of the President’s Affairs Administration of the Republic of Kazakhstan by inoculation onto Compact Dry ready-to-use chromogenic culture media and using standard microbiological methods. Colony-forming unit (CFU) counts were calculated using BactLab software, version 2.0.0. All tests were performed in parallel on deposited reference strains and field isolates.

### 4.8. Antimicrobial Activity

Testing was performed according to EUCAST recommendations, with adaptations for plant extracts. An inoculum of 1 × 10^5^ CFU/mL was spread onto the surface of agar plates (Mueller–Hinton agar for bacteria and Sabouraud dextrose agar for yeasts). The concentration of each extract, expressed as mg of dry residue per mL (mg DW/mL), was determined gravimetrically (Table 3). Sterile paper discs (6 mm diameter) were impregnated with 10 μL of undiluted extract, corresponding to 0.05–1.28 mg of dry plant material per disc (depending on the extract), and placed on the inoculated agar surface. Plates were incubated at 37 °C for bacteria and at 24 °C for Candida spp. The diameters of inhibition zones were measured using a digital caliper (±0.01 mm). Interpretive criteria were defined as follows: resistant (R), 0–10 mm; intermediate (I), 10–15 mm; and susceptible (S), 15–25 mm. All experiments were performed in triplicate.

### 4.9. Statistical Analysis

Data were analyzed using SAS software version 9.4 (SAS Institute, Cary, NC, USA). All experiments were performed with three independent replicates, and results are presented as least-squares means (LSMeans) ± standard error (SE). The model included two fixed factors: treatment group comprising individual plant extracts, phytocompositions, commercial products, the control, and microbial strain. Descriptive statistics (mean, standard deviation, minimum, maximum) were initially calculated for each group. A two-way factorial analysis of variance (ANOVA) was then performed to evaluate the main effects of treatment, microbial strain, and their interaction. Post hoc multiple comparisons among all treatment–strain combinations were conducted using Tukey’s Honestly Significant Difference (HSD) test. In addition, Dunnett’s test was applied to compare each treatment directly with the negative control. A one-way analysis of variance (ANOVA) was performed to evaluate the effect of treatment (individual plant extract) on phytochemical composition and IC_50_ values. Pearson correlation coefficients were calculated to evaluate the relationships between phytochemical composition parameters (total phenolics, phenolic acids, flavonoids, catechins) and antimicrobial inhibition zone diameters across all tested extracts, formulations, and microbial strains. Effects were considered statistically significant at *p* < 0.05.

## 5. Conclusions

Our study demonstrated that ethanol extracts from selected medicinal plants exhibit meaningful in vitro antimicrobial activity against key mastitis-causing pathogens, including *Staphylococcus aureus*, *Escherichia coli*, and *Candida albicans*. Among the investigated species, *Populus balsamifera* buds and *Humulus lupulus* showed the broadest inhibitory spectrum, while *Syzygium aromaticum* was particularly effective against reference strains. The strong antioxidant capacity of *Populus balsamifera* and *Syzygium aromaticum*, reflected in their low IC_50_ values, was consistent with their high total phenolic and flavonoid content. Phenolic compounds, particularly flavonoids and catechins, were positively correlated with antimicrobial efficacy, whereas antioxidant activity alone did not predict antimicrobial potency. Multi-component formulation F2 provided consistent broad-spectrum inhibition comparable to individual high-performing extracts, suggesting that combining complementary phytochemical profiles can be a viable strategy. Field isolates were generally more resistant than reference strains, highlighting the importance of testing against clinically relevant pathogens. Thus, our findings support the potential of phenolic-rich plant extracts as complementary or adjunct agents to conventional antibiotics in bovine mastitis management, with the broader goal of reducing antibiotic reliance and mitigating antimicrobial resistance in dairy production. However, it should be noted that all results were obtained under controlled in vitro conditions, and caution is required when extrapolating these findings to practical on-farm applications. Therefore, further studies, including in vivo evaluations in dairy cows and safety assessments, are necessary to confirm the efficacy and applicability of these plant-derived preparations for mastitis management.

## Figures and Tables

**Table 1:** Phytochemical composition of ethanol extracts from 10 medicinal plant species.

Extract	Total Phenolic Compounds, mg GAE g^−1^ Extract	Phenolic Acids, mg CAE g^−1^ Extract	Flavonoids, mg QE g^−1^ Extract	Catechins, mg CE g^−1^ Extract
Balsam poplar buds (*Populus balsamifera*)	43.0 ± 0.2 ^a^	12.6 ± 0.1 ^a^	34.5 ± 0.2 ^a^	0.9 ± 0.01 ^b^
Clove (*Syzygium aromaticum*)	31.8 ± 0.2 ^b^	3.5 ± 0.1 ^b^	22.8 ± 0.2 ^b^	0.3 ± 0.01 ^c^
Wormwood (*Artemisia absinthium*)	12.1 ± 0.2 ^c^	2.2 ± 0.1 ^c^	7.9 ± 0.2 ^d^	<0.0001 ^e^
Broadleaf Plantain (*Plantago major*)	11.7 ± 0.2 ^c^	2.5 ± 0.1 ^c^	8.5 ± 0.2 ^c^	1.0 ± 0.01 ^a^
Yarrow (*Achillea millefolium*)	7.2 ± 0.2 ^d^	2.2 ± 0.1 ^c^	5.1 ± 0.2 ^e^	<0.0001 ^e^
Hops (*Humulus lupulus*)	4.0 ± 0.2 ^e^	1.6 ± 0.1 ^d^	2.2 ± 0.2 ^f^	<0.0001 ^e^
Polypore (*Fomes fomentarius*, *ex Populus*)	2.9 ± 0.2 ^f^	0.5 ± 0.1 ^f^	1.5 ± 0.2 ^g^	0.1 ± 0.01 ^d^
Coriander (*Coriandrum sativum*)	2.5 ± 0.2 ^f^	1.3 ± 0.1 ^de^	1.2 ± 0.2 ^g^	<0.0001 ^e^
Milk Thistle (*Silybum marianum*)	3.7 ± 0.2 ^e^	1.1 ± 0.1 ^e^	2.5 ± 0.2 ^f^	0.1 ± 0.01 ^d^
Chamomile (*Matricaria chamomilla*)	7.3 ± 0.2 ^d^	2.5 ± 0.1 ^c^	4.7 ± 0.2 ^e^	<0.0001 ^e^
Effect of extract, *p*-value	<0.001	<0.001	<0.001	<0.001

Data are presented as least-squares means (LSMeans) ± standard error (SE) of three replicates. GAE = gallic acid equivalents; CAE = caffeic acid equivalents; QE = quercetin equivalents; CE = catechin equivalents. Values <0.0001 mg/g indicate concentrations below catechin detection limit (LOD = 0.0001 mg/g). LSMeans within a column followed by different superscript letters differ significantly at *p* < 0.05.

**Table 2:** Pearson correlation coefficients between antimicrobial activity and phenolic composition.

Variable	*Candida* Field	*Candida* ATCC	*E. coli* Field	*E. coli* ATCC	Total Field	*S. aureus* ATCC	Total Phenolics	Phenolic Acids	Flavonoids	Catechins
IC_50_	0.079	0.160	0.310	0.048	−0.257	0.193	−0.410	−0.361	−0.383	−0.283
*Candida* field		0.848 **	−0.612	0.283	0.365	0.731 *	0.638 *	0.478	0.629	0.396
*Candida* ATCC			−0.460	0.235	0.475	0.674 *	0.701 *	0.584	0.718 *	0.343
*E. coli* field				0.367	−0.297	−0.727 *	−0.574	−0.668 *	−0.615	−0.604
*E. coli* ATCC					−0.030	−0.111	0.117	−0.318	0.048	−0.364
Total field						0.516	0.718 *	0.573	0.743 *	0.833 **
*S. aureus* ATCC							0.572	0.525	0.614	0.711 *
Total phenolics								0.883 **	0.994 **	0.630
Phenolic acids									0.901 **	0.628
Flavonoids										0.663 *

Levels of significance: * *p* < 0.05; ** *p* < 0.01.

**Table 3:** Inhibition zone diameters (mm) of plant extracts, F1–F2, C1–C2 and standard antibiotics against deposited reference strains and experimental mastitis field isolates.

Extract	Concentration (mg DW/mL)	*Candida* Field	*Candida* ATCC	*E.coli* Field	*E.coli* ATCC	Total Field	*S.aureus* ATCC
*Populus balsamifera* buds	68	15.1 ± 2.9	18.1 ± 2.9	4.7 ± 2.9	6.7 ± 2.9	13.8 ± 2.9	19.1 ± 2.9
*Syzygium aromaticum*	128	12.5 ± 2.9	14.5 ± 2.9	13.5 ± 2.9	17.5 ± 2.9	13.7 ± 2.9	13.5 ± 2.9
*Humulus lupulus*	43	18.2 ± 2.9	17.6 ± 2.9	9.0 ± 2.9	13.2 ± 2.9	8.2 ± 2.9	18.4 ± 2.9
*Silybum marianum*	46	9.6 ± 2.9	7.7 ± 2.9	10.8 ± 2.9	8.3 ± 2.9	13.4 ± 2.9	19.0 ± 2.9
*Coriandrum sativum*	33	5.2 ± 2.9	10.8 ± 2.9	17.1 ± 2.9	10.7 ± 2.9	10.8 ± 2.9	12.7 ± 2.9
*Plantago major*	63	8.1 ± 2.9	3.6 ± 2.9	13.8 ± 2.9	11.6 ± 2.9	8.7 ± 2.9	9.1 ± 2.9
*Fomes fomentarius*	24	9.2 ± 2.9	7.8 ± 2.9	12.4 ± 2.9	9.8 ± 2.9	11.1 ± 2.9	10.1 ± 2.9
*Artemisia absinthium*	7	3.2 ± 2.9	8.1 ± 2.9	13.6 ± 2.9	9.3 ± 2.9	9.4 ± 2.9	8.9 ± 2.9
*Achillea millefolium*	5	2.3 ± 2.9	0.0 ± 2.9	10.1 ± 2.9	9.2 ± 2.9	8.9 ± 2.9	12.7 ± 2.9
*Matricaria chamomilla*	49	2.9 ± 2.9	2.0 ± 2.9	15.6 ± 2.9	10.2 ± 2.9	9.0 ± 2.9	6.5 ± 2.9
F1		3.2 ± 2.9	5.6 ± 2.9	5.1 ± 2.9	4.0 ± 2.9	7.6 ± 2.9	9.4 ± 2.9
F2		12.6 ± 2.9	17.2 ± 2.9	14.8 ± 2.9	15.4 ± 2.9	0.0 ± 2.9	15.4 ± 2.9
CP1		4.6 ± 2.9	14.9 ± 2.9	11.0 ± 2.9	7.4 ± 2.9	6.2 ± 2.9	15.8 ± 2.9
CP2		8.9 ± 2.9	28.8 ± 2.9	11.1 ± 2.9	6.8 ± 2.9	6.4 ± 2.9	10.5 ± 2.9
Ethanol control (%)	70	0.0 ± 2.9	0.0 ± 2.9	0.0 ± 2.9	0.0 ± 2.9	0.0 ± 2.9	0.0 ± 2.9
Ampicillinum (mg/mL)	2.5	-	-	-	-	14.5 ± 2.9	-
Carbenicillin (mg/mL)	10	-	-	10.5 ± 2.9	-	-	-
Cefalotin (mg/mL)	5	-	-	-	-	0.0 ± 2.9	-
Imipenem (mg/mL)	10	-	-	11.6 ± 2.9	-	-	-
Ketoconazole (mg/mL)	2	8.9 ± 2.9	-	-	-	-	-
Fluconazole (mg/mL)	1.5	0.0 ± 2.9	0.0 ± 2.9	-	-	-	-
Nystatin (IU/mL)	2.5	0.0 ± 2.9	0.0 ± 2.9	-	-	-	-

Data are presented as LSMeans ± SE of three replicates. A dash (“-”) in the table rows indicates that testing for the presence/absence of the specified microorganisms was not performed.

**Table 4:** Characteristics of the plant materials used in the study.

Species (with Authority)	Family	Plant Part Used	Collection Site/Origin	Coordinates	Pharmacopoeia Data/Origin	POWO Link (All Accessed on 18 March 2026)
*Coriandrum sativum* L.	Apiaceae	Seeds	Uzbekistan	*	SP RK, т. II	https://powo.science.kew.org/taxon/urn:lsid:ipni.org:names:840760-1
*Matricaria chamomilla* L.	Asteraceae	Flowers	Akmola region, Kazakhstan	51.246724, 71.769323	SP RK, т. II. c. 704–705.	https://powo.science.kew.org/taxon/urn:lsid:ipni.org:names:154715-2
*Plantago* *major* L.	Plantaginaceae	Leaves	Akmola region, Kazakhstan	51°18′29.7′′N 70°42′23.6′′E	SP RK, т. III	https://powo.science.kew.org/taxon/urn:lsid:ipni.org:names:77227281-1
*Silybum marianum* (L.) Gaertn.	Asteraceae	Seeds	Akmola region, Kazakhstan	46.100284, 80.642703	SP RK, т. II.	https://powo.science.kew.org/taxon/urn:lsid:ipni.org:names:249211-1
*Populus balsamifera* L.	Salicaceae	Buds	Akmola region, Kazakhstan	51.291016, 70.682656	SP RF, 13th ed.; Vol. III. FS.2.5.0042.15.	https://powo.science.kew.org/taxon/urn:lsid:ipni.org:names:776600-1
*Achillea* *millefolium* L.	Asteraceae	Aerial parts	Akmola region, Kazakhstan	43.254748, 77.189459	SP RK, т. II	https://powo.science.kew.org/taxon/urn:lsid:ipni.org:names:2294-2
*Artemisia* *absinthium* L.	Asteraceae	Aerial parts	Akmola region, Kazakhstan	51.310994, 70.654803	SP RK, т. II	https://powo.science.kew.org/taxon/urn:lsid:ipni.org:names:300106-2
*Syzygium aromaticum* (L.) Merr. & L.M.Perry	Myrtaceae	Flower buds	Commercial botanical material	**	SP RF, 13th ed.; Vol. III. FS.0.0.0000	https://powo.science.kew.org/taxon/urn:lsid:ipni.org:names:601421-1
*Humulus* *lupulus* L.	Cannabaceae	Cones	Akmola region, Kazakhstan	51.294751, 70.746605	SP RF, 13th ed.; Vol. III. FS.2.5.0046.15	https://powo.science.kew.org/taxon/urn:lsid:ipni.org:names:303502-2

* NATRULI COMPANY LLC, 27 Korneeva Street, Premises 1, Domodedovo, Russian Federation. ** AL JAMEEL INTERNATIONAL FOOD INDUSTRY CO LLC, Box 64369UAE, Deira Al Ras Area P.O., National Industries Park, Dubai, UAE.

**Table 5:** Characteristics of the fungal material used in the study.

Species (with Authority)	Family	Plant Part Used	Collection Site/Origin	Coordinates	Pharmacopoeia Data/Origin	MYCOBANK Link (Accessed on 18 March 2026)
*Fomes fomentarius* (L.) Fr.	Polyporaceae	Basidiocarp (fruiting body)	Akmola region, Kazakhstan	52.04587, 71.174895	MycoBank, fungal taxonomic database	https://www.mycobank.org/page/Name%20details%20page/10408?utm_source=chatgpt.com

**Table 6:** Composition of multi-component plant extract formulations.

Formulation	Plant Extracts (Mass Fraction, %)	Total Extract Fraction (%)	Other Ingredients (% *w*/*w*)	Water (% *w*/*w*)	Glycerin (% *w*/*w*)	Xanthan Gum (% *w*/*w*)
F1	*Humulus lupulus* (7.07), *Artemisia absinthium* (6.06), *Calendula officinalis* (6.06), *Plantago major* (6.06), *Populus balsamifera* buds (5.05), *Achillea millefolium* (4.04)	34.34	–	55.35	10.10	0.2
F2	*Coriandrum sativum* (4.0), *Syzygium aromaticum* (4.0), *Fomes fomentarius* (4.0), *Populus balsamifera* buds (3.0), *Matricaria chamomilla* (3.0)	18.0	Flax oil (10.0), Beeswax (1.0)	60.0	10.0	1.0

## Data Availability

The original contributions presented in this study are included in the article. Further inquiries can be directed to the corresponding author.

## Supplementary Materials

The following supporting information can be downloaded at https://www.mdpi.com/article/10.3390/molecules31071089/s1, Figure S1. Balsam poplar buds (*Populus balsamifera*); Figure S2. Hops (*Humulus lupulus*); Figure S3. Yarrow (*Achillea millefolium*); Figure S4. Milk Thistle (*Silybum marianum*); Figure S5. Wormwood (*Artemisia absinthium*); Figure S6. Broadleaf Plantain (*Plantago major*); Figure S7. Coriander (*Coriandrum sativum*); Figure S8. Clove (*Syzygium aromaticum*); Figure S9. Chamomile (*Matricaria chamomilla*); Figure S10. Polypore (*Fomes fomentarius, ex Populus*).
