# Supplementary material for: In Vitro Antimicrobial Potential of Medicinal Plant Extracts and Their Combinations Against Mastitis-Causing Bacteria in Dairy Cows
Source: Molecules. 2026 Mar 26;31(7):1089. doi: 10.3390/molecules31071089 (PMC13074613; doi:10.3390/molecules31071089)
Supplement: Supplementary file 1 [file molecules-31-01089-s001.zip › molecules-4203781-supplementary.pdf]

Supplementary Figures S1-S10. Raw plant materials collected for extraction

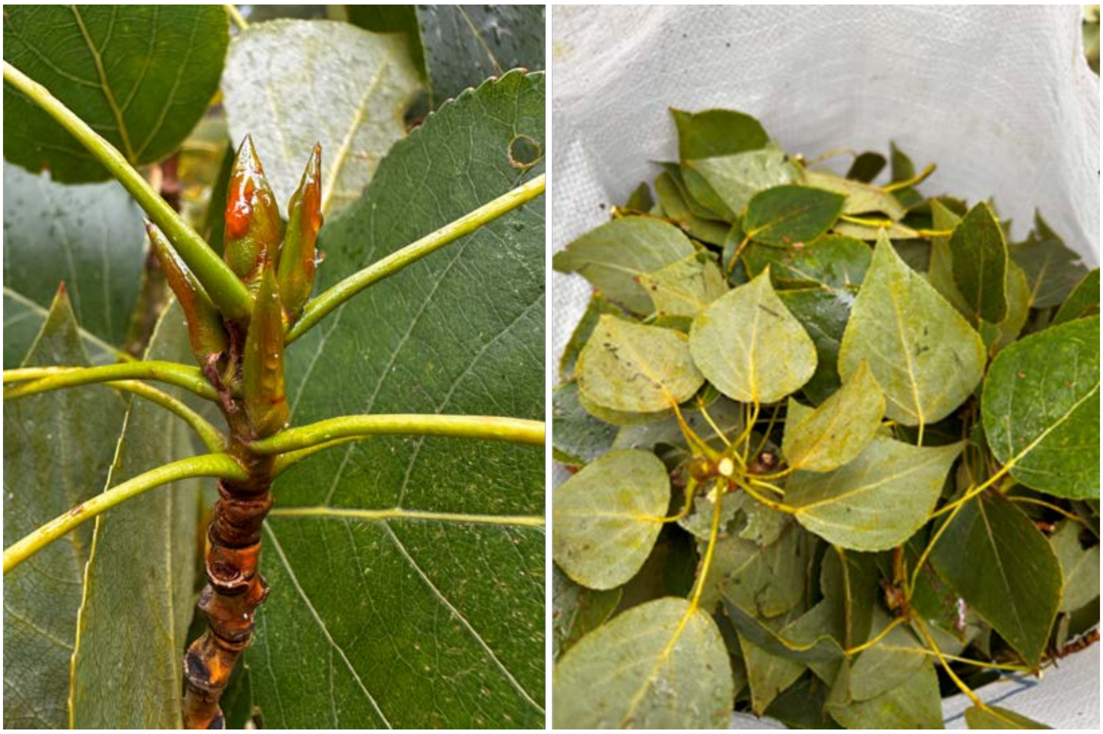

Figure S1. Balsam poplar buds *(Populus balsamifera)*

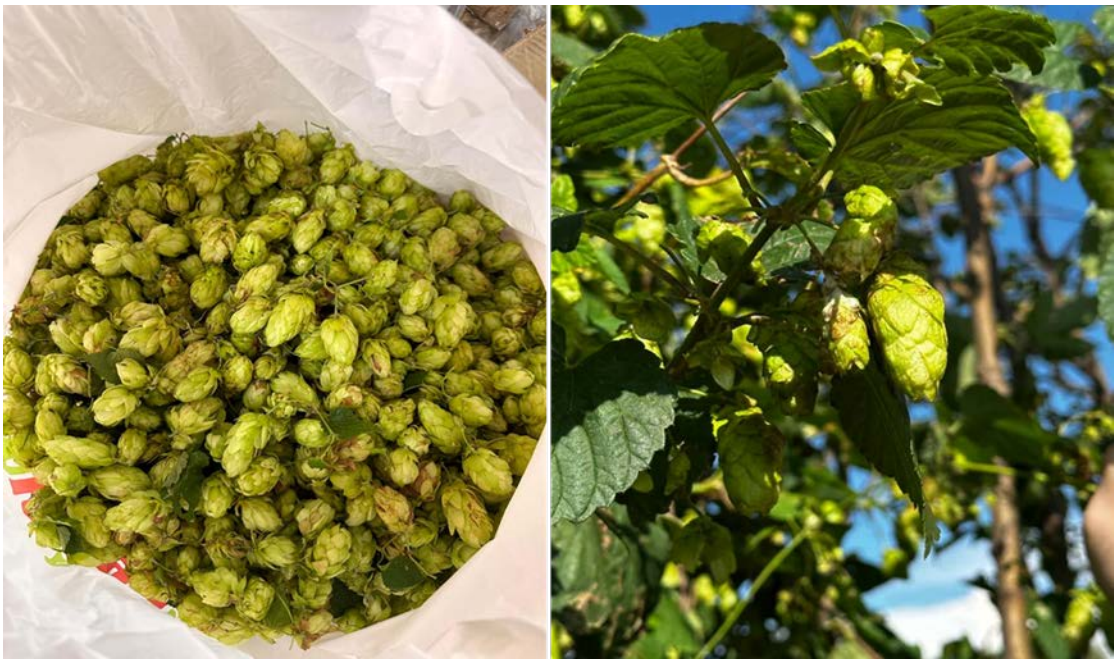

Figure S2. Hops *(Humulus lupulus)*

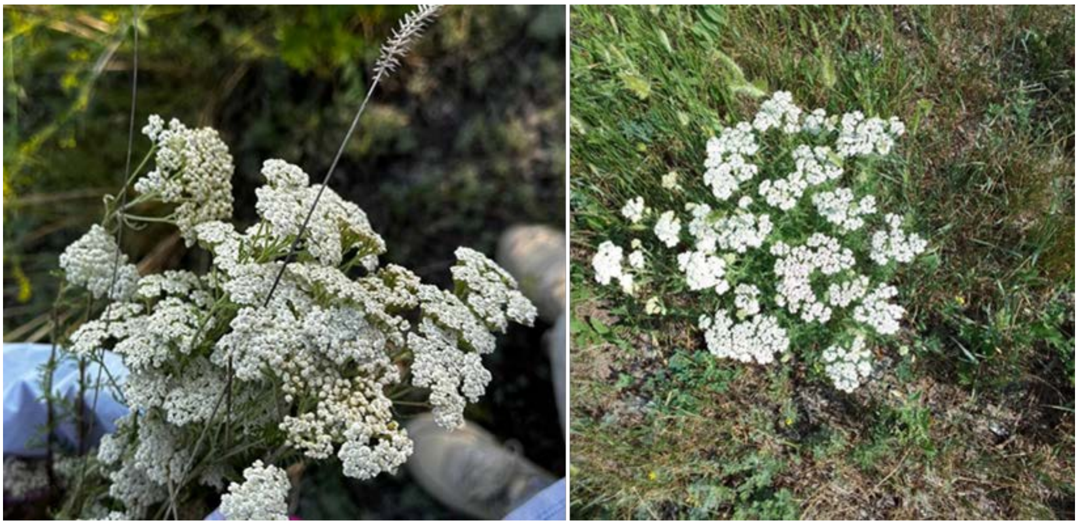

Figure S3. Yarrow *(Achillea millefolium)*

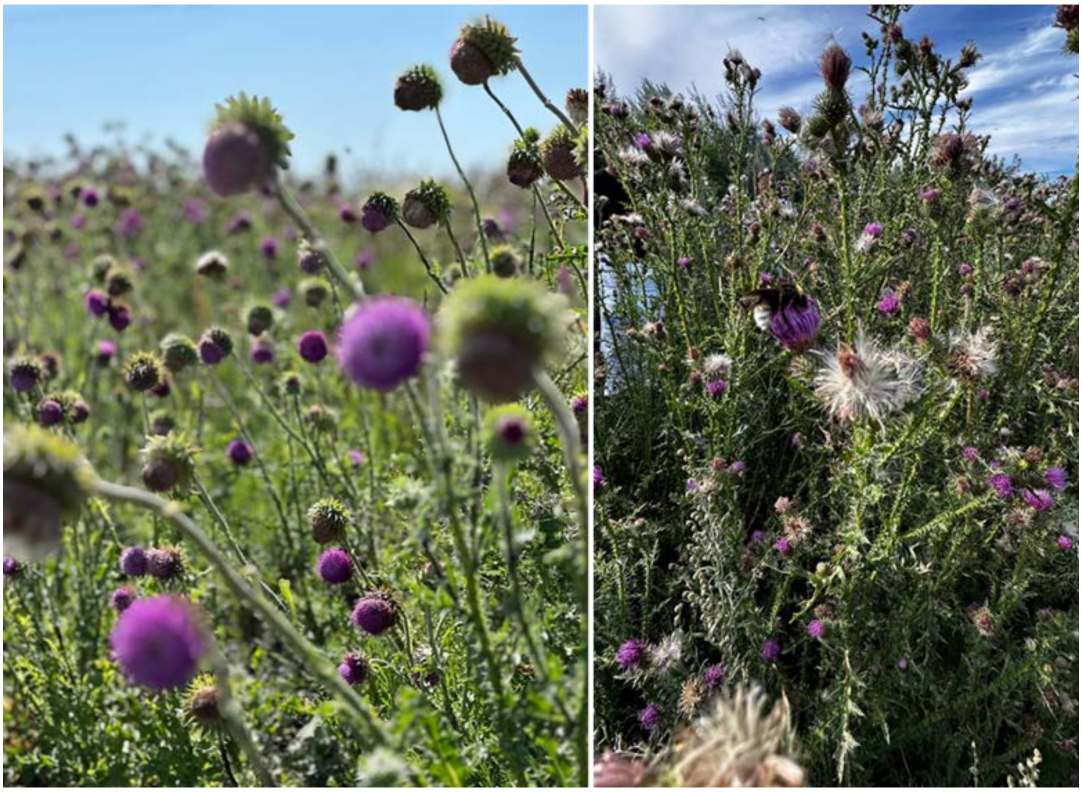

Figure S4. Milk Thistle *(Silybum marianum)*

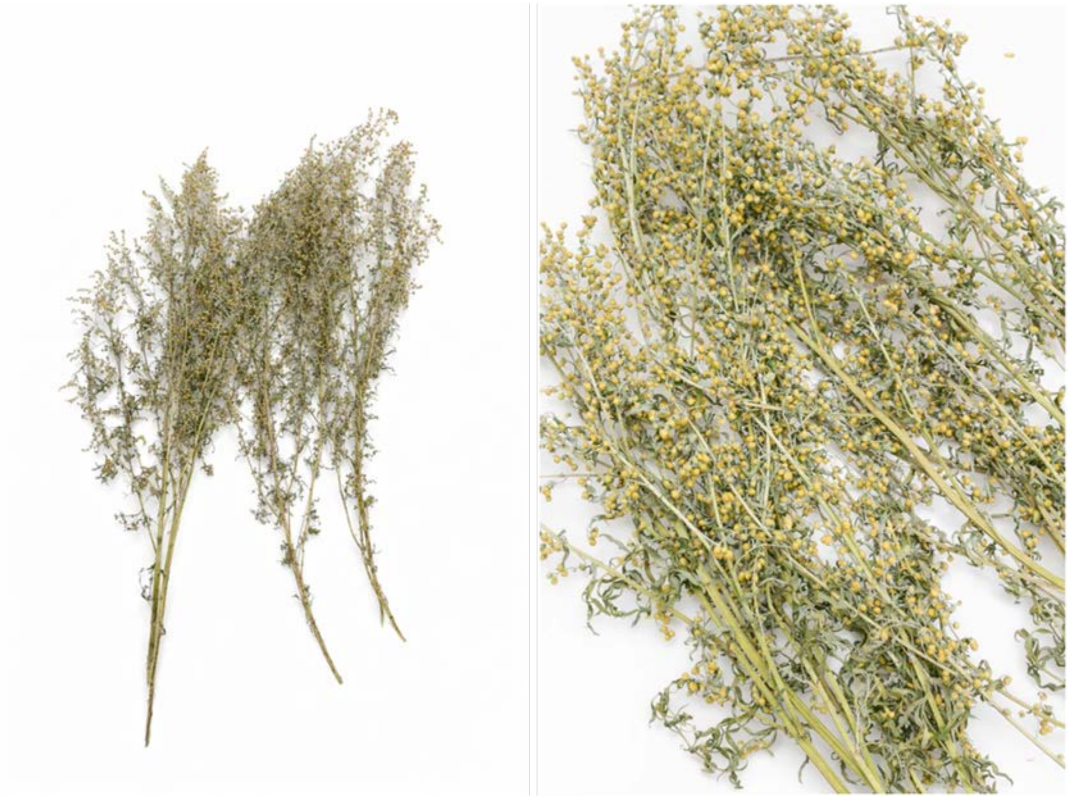

Figure S5. Wormwood *(Artemisia absinthium)*

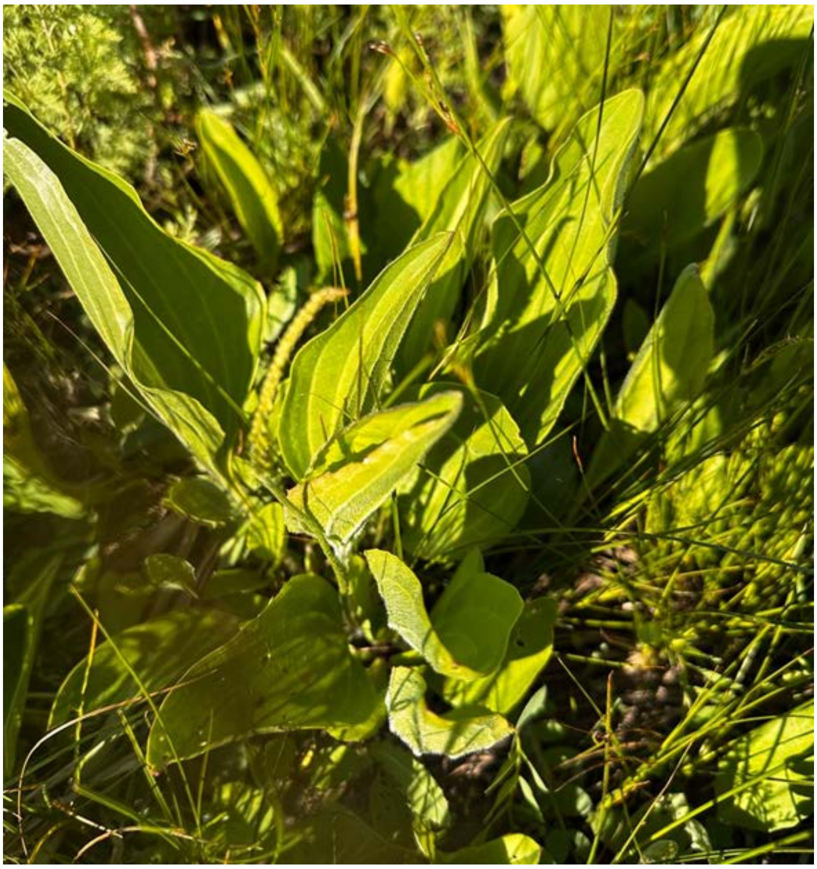

Figure S6. Broadleaf Plantain *(Plantago major)*

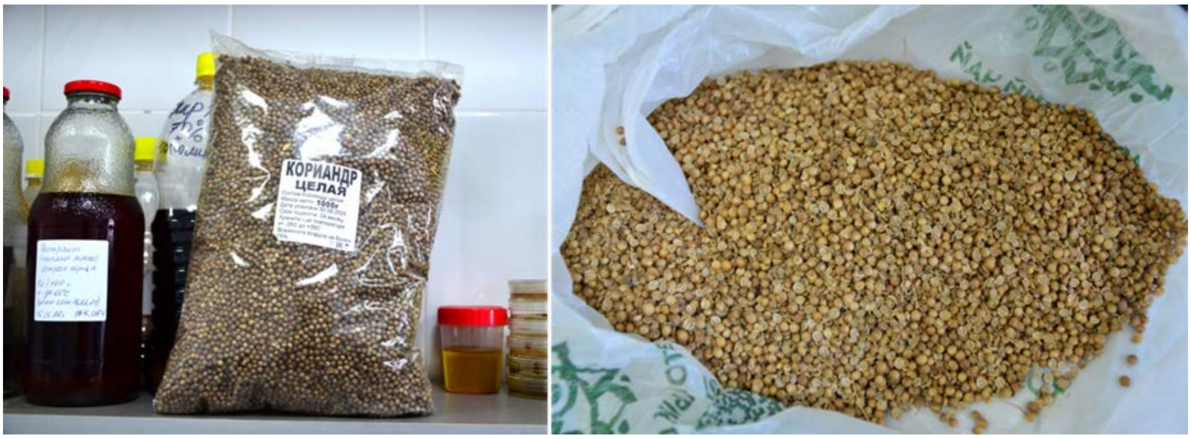

Figure S7. Coriander *(Coriandrum sativum)*

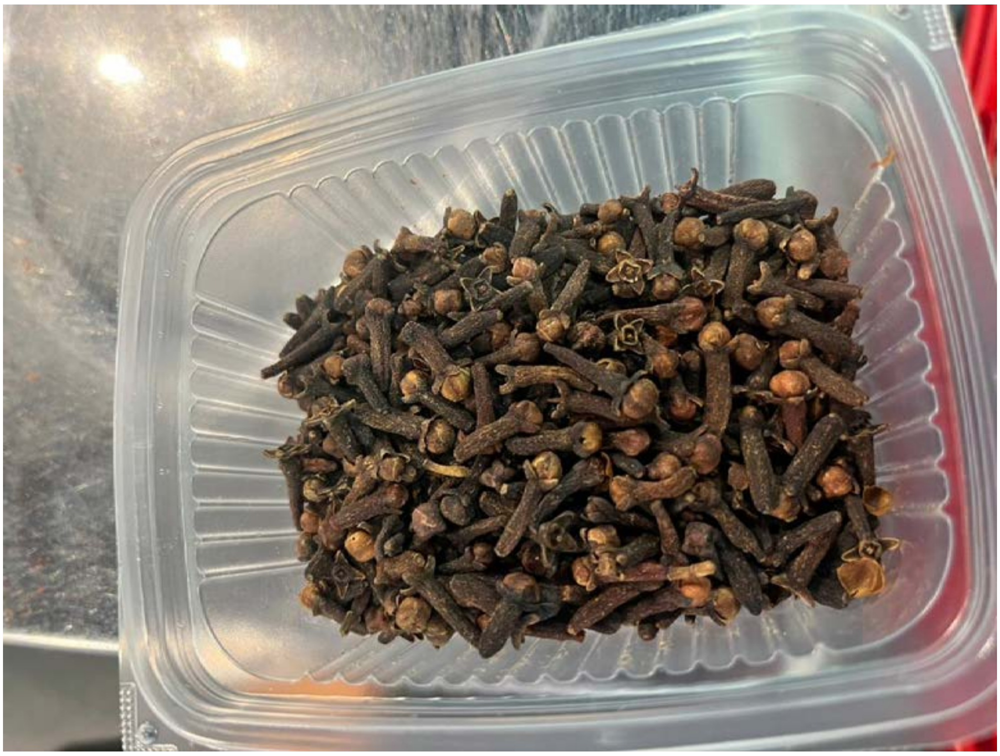

Figure S8. Clove *(Syzygium aromaticum)*

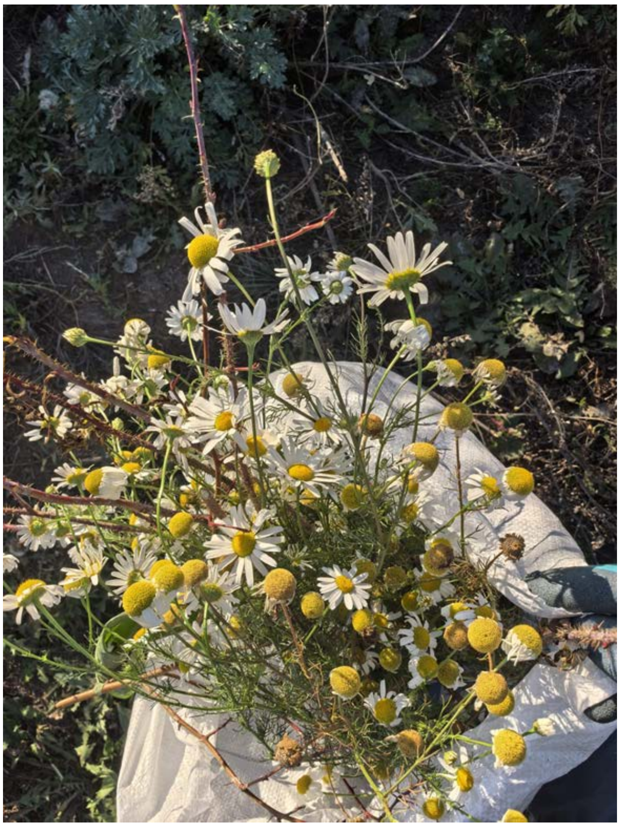

Figure S9. Chamomile (*Matricaria chamomilla*)

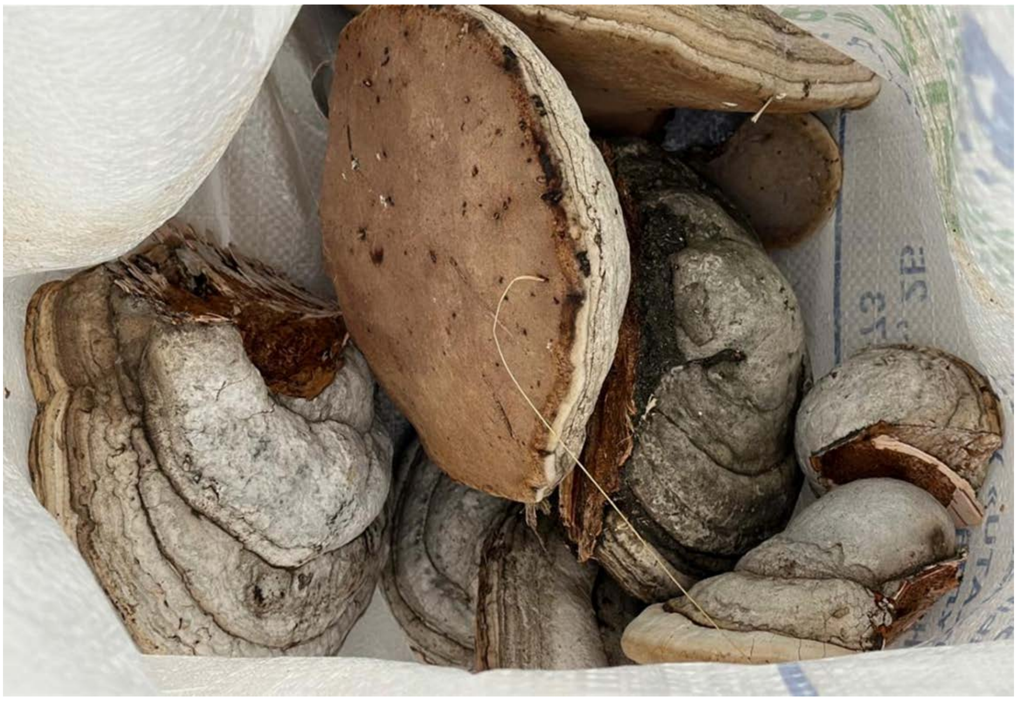

Figure S10. Polypore *(Fomes fomentarius, ex Populus)*
